# Comparative proteomic analysis of mitochondrial proteins from maize CMS‐C sterile, maintainer and restorer anthers

**DOI:** 10.1002/tpg2.20022

**Published:** 2020-05-22

**Authors:** Huaisheng Zhang, Bin Wang, Bing Li, Yanan Lin, Huili Yang, Dong Ding, Yadong Xue, Jihua Tang

**Affiliations:** ^1^ College of Agronomy, National Key Laboratory of Crop Science in Wheat and Maize Henan Agricultural University Zhengzhou China; ^2^ College of Agronomy Henan Science and Technology University Luoyang China

## Abstract

The maize C system of cytoplasmic male sterility (CMS) and its fertility restoration gene *Rf4* have been widely used for maize hybrid production; however, the underlying mechanism is still uncertain. The sterility factor functions in mitochondria, where it interacts directly or indirectly with the restorer. Mitoproteomics can capture all participants involved in CMS and restoration at the organelle level. In the present study, we identified and quantified anther mitochondrial proteins from CMS, maintainer and restorer lines. We obtained 14,528 unique peptides belonging to 3,369 proteins. Comparative analysis of 1840 high‐confidence proteins revealed 68 were differentially accumulated proteins likely involved in CMS or its restoration within mitochondria. These proteins were mainly associated with fatty acid metabolism, amino acid metabolism and protein‐processing pathways. These results suggest that an energy deficiency caused by the sterility factor hinders other proteins or protein complexes required for pollen development through nuclear‐mitochondrial interaction. The restorer factor may boost the energy generation by activating alternative metabolic pathways and by improving the post‐translation processing efficiency of proteins in energy‐producing complexes to restore pollen fertility. Our findings may aid detailed molecular analysis and contribute to a better understanding of maize CMS‐C restoration and sterility.

**Abbreviations**
CMS: cytoplasm male sterility
DAP: differentially accumulated protein
iTRAQ: isobaric tags for relative and absolute quantification

## 1 INTRODUCTION

Plant cytoplasmic male sterility (CMS), the inability to produce functional pollen, is a widespread plant reproductive feature (Chen & Liu, 2014). This maternally inherited trait has been extensively used in hybrid seed production to improve crop yields and exploit heterosis.

To generate high‐yield hybrid seeds, elucidation of the CMS mechanism is therefore necessary. Plant CMS arises from the recombinations of mitochondrial DNA, which leads to aberrant chimeric genes (Chase, 2007). The deleterious effects of CMS are eliminated by specific nuclear gene(s) called restorer‐of‐fertility (*Rf*) through the processing of CMS‐associated transcripts or peptides (Barkan & Small, 2014; Chen & Liu, 2014; Hu et al., 2014). Current models invoked to explain CMS mechanism include cytotoxicity, energy deficiency, aberrant programmed cell death and retrograde regulation models with the corresponding *Rf* genes functioning at the genomic, posttranscriptional, translational, posttranslational or metabolic levels to restore male fertility (Chen & Liu, 2014).

Maize CMS is categorized into three major types (CMS‐T, CMS‐S, and CMS‐C) according to the pattern of fertility restoration (Beckett, 1971). CMS‐T is associated with the mitochondrial gene *T‐urf13* (Dewey, Levings, & Timothy, 1986), which encodes a protein that is inserted into the inner mitochondrial membrane (Hack, Lin, Yang, & Horner, 1991; Korth, Kaspi, Siedow, & Levings, 1991). *Rf1* and two other restorers, *Rf8* and Rf*, mediate the differential processing of *T‐urf13* transcripts. In combination with one of these three restores, *Rf2* restores the fertility of T‐cytoplasm maize (Wise, Gobelman‐Werner, Pei, Dill, & Schnable, 1999), probably by ameliorating the mitochondrial lesion associated with T‐URF13 or by scavenging and detoxifying acetaldehyde generated in energy deficit (Cui, Wise, & Schnable, 1996). The polycistronic chimeric orf, *orf355‐orf77*, is the cause of CMS‐S in maize (Wen & Chase, 1999). The cleavage of this transcript in an *Rf3* background, which further causes the degradation of the resulting 1.6‐kb transcript, leads to fertility restoration (Xiao, Zhang, & Zheng, 2006). Considering the structures and the processing pattern in the restorer lines of the CMS transcripts, the cytotoxicity model may partially explain the CMS mechanism in CMS‐T and CMS‐S maize. Three chimeric mitochondrial genes, *atp6c*, *atp9‐2* and *cox2‐2*, have been proposed as the candidate CMS‐C gene, but no direct evidence exists that they are responsible for the male sterile phenotype (Allen et al., 2007; Dewey, Timothy, & Levings, 1991). In addition to the two duplicate restorer genes, *Rf4* and *Rf5*, *Rf6*, Rf*‐A619 and some other quantitative trait loci are involved in partial or full restoration of male fertility for CMS‐C (Kohls, Stamp, Knaak, & Messmer, 2011; Sisco, 1991; Tang et al., 2001; Yongming et al., 2016). Furthermore, an inhibitor gene of *Rf5*, *Rf‐I*, was mapped to chromosome 7 (Hu et al., 2006). Because of limited knowledge of the CMS‐C‐causing gene and restorer genes, the mechanisms of sterility and fertility restoration are still unclear.

**Core Ideas**

- Changes of proteins within mitochondria may be useful for interpreting the mechanism of CMS.
- Differences in protein expression supported the energy deficiency hypothesis for maize CMS‐C.
- A lines comparison suggests the restorer gene initiates alternative metabolic pathways to meet energy requirements.
- Restorer gene improves the efficiency of post‐translation processing to promote its maturation and assembly.

Genome‐scale expression profiling has provided some clues concerning the CMS mechanism in CMS‐C maize. For instance, 20 genes and 25 proteins, identified by comparison of a sterile line and its maintainer line using suppression subtractive hybridization and 2‐D electrophoresis, respectively, have been found to be involved in energy metabolism, molecular chaperoning, and cell death during tetrad, uninucleate, and binucleate stages (Huang et al., 2012). Additional pathways, including those related to polyamine metabolic process, cutin, suberin and wax biosynthesis, Fatty acid elongation, biosynthesis of unsaturated fatty acids and proline metabolism were detected through comparative transcriptome analysis of anthers from pollen mother cell and mononuclear stages (Li et al., 2017b). Although high transcript abundance has been generally believed to correspond to a high protein level, recent research has revealed that the correlation can be as low as 40% (Vogel & Marcotte, 2012). A proteomics approach can lay a foundations for better comprehension of CMS‐C mechanisms, and the method known as isobaric tags for relative and absolute quantification (iTRAQ) has relatively higher sensitivity and reproducibility features compared with 2‐D electrophoresis technology (Jiang et al., 2014; Mateos et al., 2015; Wang et al., 2017). In the present study, the iTRAQ‐based proteomics method was applied to identify mitochondrial‐level differentially expressed elements associated with CMS and fertility restoration among maintainer, sterile and fertile maize lines. Our results may provide insights into the mechanism of maize CMS‐C from a mitochondrial perspective.

## 2 MATERIALS AND METHODS

### 2.1 Plant materials

The male sterile line C^es^87‐1*^rfrf^* (**indicated as letter A**), (“C” means the C cytotype, “es” means subgroup of C‐type CMS) was obtained by successive backcrossing to N87‐1*^rfrf^* (**indicated as letter B**), (“N” means the normal cytotype) for more than eight generations. C^es^87‐1*^rfrf^* was crossed with a restorer line Fengke1*^Rf4Rf4Rf5Rf5^*, then back‐crossed to C^es^87‐1*^rfrf^* for more than six generations. During back‐crossing, molecular markers were tested to select a fertile near‐isogenic line (NIL) with *Rf4* only in the 87‐1 nuclear background. This NIL was designated C^es^87‐1*^Rf4Rf4^* (**indicated as letter R**). The three lines were planted at the farm of Henan Agricultural University (Zhengzhou, Henan Province, China) during Spring‐Summer 2014. Forty individual plants in two‐row plots for each line were planted at every seven days for continuous sampling. The upper three anthers develop 1 day ahead of the lower three anther in the same spikelet (Kelliher & Walbot, 2011). The anthers from upper floret were collected in order to eliminate the developmental difference. After careful development staging, anthers at the dyad (**indicated as digit 3**) and tetrad (**indicated as digit 4**) stages from more than 20 individual plants were pooled for each replicate. For convenience, we used a combination of one letter and one digit represents a specific sample at a specific stage, for example, “**A3**” means the dyad anthers from sterile lines.

### 2.2 Mitochondria fractionation

Anthers about 1–2 g were ground in 15 ml prechilled grinding buffer (0.05 mol/L Tris‐Cl, 0.5 mol/L Sucrose, 0.005 mol/L EDTA.Na_2_, 0.1% BSA, 0.005 mol/L β‐ME, 1% PVP, pH7.5). The grindings were filtered into 50 ml ice‐cold centrifuge tube through 6 layers of cheesecloth and centrifuged at 1,200 *g* and 4 °C for 15 min. The supernatant was transferred to a new 50 ml centrifuge tube and centrifuged at 2,000 *g* and 4 °C for 20 min to pellet mitochondria. The crude mitochondria pellet was suspended in the suspension buffer (0.05 mol/L Tris‐Cl, 0.3 mol/L sucrose, 0.01 mol/L MgCl_2_, 1% PVP, pH7.5) and was centrifuged at 12,000 g and 4 °C for 15 min after mixing. 12.5 μg gDNase I were added and incubated at room temperature for 1–2 hours. The reaction was terminated by adding 2.5 ml washing buffer (0.01 mol/L Tris‐Cl, 0.6 mol/L Sucrose, 0.02 mol/L EDTANa_2_. pH7.2) and centrifuged at 16,000 g and 4 °C for 15 min to collect mitochondria. The supernatant was discarded and the pellet was washed with 5 ml washing buffer and then centrifuged at 16,000 g and 4 °C for 15 min. The washing step was repeated two more times.

### 2.3 Protein extraction and quantification

Protein extraction was performed according to the protocol described in (Wiśniewski, Zougman, Nagaraj, & Mann, 2009). Proteins were quantified with the Bradford assay using BSA as the calibrant (Bradford, 1976). The quality of the protein sample was assayed by SDS‐PAGE.

### 2.4 Protein digestion and iTRAQ labelling

DTT was added to final concentration of 100 mM in each 200 μg protein sample. After boiling for 5 min and cooling to room temperature, 200 μl UA buffer (8 mol/L urea, 150 mmol/L TrisHCl pH8.0) were added to eliminate DTT and other small elements. The mixture was centrifuged at 14,000 *g* for 15 min in a 10 kb ultrafiltration tube. The filtration was repeated once with another 200 μl UA buffer. After discarding the filtrate, 100 μl IAA (50 mM iodoacetamide in UA buffer) was added to seal reduced cysteine residues. The samples were oscillated at 600 rpm for 1 min and then developed for 30 min in darkness. The filters were abluted with 100 μl UA buffer at 14,000 *g* for 10 min three times and then washed with 100 μl DS buffer (dissolution buffer) twice. Finally, 40 Trypsin buffer (4 μg Trypsin in 40 μl DS buffer) was added into the protein suspensions for peptide digestion overnight at 37°C, and the digested peptides were collected as a filtrate at 14,000 *g* for 10 min. The peptide content was measured by UV light spectral density at 280 nm (Wiśniewski et al., 2009). Each peptide solution (50 μg peptide mixture) were labeled with iTRAQ 8‐plex Multiplex kits (AB SCIEX, USA) according to the manufacturer's protocol. The samples A3‐1, A4‐1, B3‐1, B4‐1 and R4‐1 from replication 1 were labeled with the iTRAQ tags 114, 115, 116, 117 and 118. The samples A3‐2, A4‐2, B3‐2, B4‐2 and R4‐2 from replication 2 were labeled with the tags 114, 115, 116, 117 and 118. After labeling, equivalent volumes of each sample from replication 1 and replication 2 were pooled, respectively.

### 2.5 LC‐MS/MS analysis by Q exactive

The two samples were separated using Thermo Scientific Easy‐nLC system, respectively. Columns were balanced with 95% Buffer A (0.1% formic acid solution in water). The mixtures was first loaded onto Thermo Scientific EASY column (2 cm × 100 μm, 5 μm C18) at a flow rate of 250 nl/min, then separated by Thermo scientific EASY analytical column (75 μm × 100 mm 3 μm‐C18). The 240‐min gradient was run starting from 0% Buffer B (84% ACN in 0.1% formic acid) to 55% Buffer B for 220 min, 55–100% Buffer B for 8 min and 100% Buffer B from 228 min to 240 min.

Mass spectrometry was performed with Q‐Exactive mass spectrometer (Thermo Fisher Scientific, San Jose, CA, USA). Data was generated using the parameter settings of the machine as follows: MS spectra of 300–1,800 m/z at a resolution of 7,000 at 200 m/z were collected with an automatic gain control target fixed at 3 × 10^6^ ions and a maximum injection time of 10 ms. The dynamic exclusion duration was set to 40.0s. The top ten precursor ions after each full scan were collected for fragmentation by higher energy collisional dissociation at a 2‐m/z isolation window and with normalized collision energies of 30 eV. For MS/MS scan, the resolution was 17,500 at 200 m/z with a maximum injection time of 60s. The underfill ratio was set to 0.1%.

### 2.6 Database identification and iTRAQ quantification

Two raw data files were submitted to Mascot2.2 server through Proteome Discover1.4 (Thermo) for raw peptide and protein identification against the UniProtKB/Swiss‐Prot database (release of 2015‐01‐07, species: *Zea mays*, 85,072 entries). The parameters for searching were as follows: monoisotopic mass, trypsin as the enzyme with allowance for two missed cleavages, Carbamidomethyl (C), iTRAQ8plex (N‐term) and iTRAQ8plex (K) labeling as fixed modifications, the oxidation (M) and iTRAQ8plex (Y) were specified as variable modifications. The fragment mass tolerance and peptide mass tolerance were set to 0.1 Da and ±20 ppm, respectively. The false discovery rate (FDR ≤ 0.01) was estimated by an automatic decoy database search strategy in Mascot. For protein quantification, the peak intensities of the iTRAQ reporter ions released in each of the MS/MS spectra were extracted using Proteome Discover (Version 1.4). The median of only unique peptides of the protein was used for protein ratio calculation. The median ratio was set to weight and normalize quantitative protein. Only fold changes of > 1.2 and significance threshold P < .05 (with 95% confidence) were considered as significantly changed proteins.

### 2.7 Mitoproteomic analysis

GO ontology was performed using clusterProfiler (Yu, Wang, Han, & He, 2012). Pathways associated with differentially accumulated proteins were extracted through KEGG_PATHWAY, PANTHER_PATHWAY and REACTOME_PATHWAY (Huang et al., 2007).

### 2.8 RNA isolation and quantitative real‐time PCR

RNA was isolated with the RNAprep Pure Plant Kit (TIAN TEN Cat#DP411, China) following the manufacturer's manual. The first strand cDNA was obtained through reverse transcription using PrimeScript RT Reagent Kit with gDNA Eraser (Perfect Real Time) (Cat#RR047A, Japan) according to the manufacturer's instructions and the qRT‐PCR were carried out using the ChamQ Universal SYBR qPCR Master Mix (Cat#Q711‐02, Vazyme, China). The qTR‐PCR was performed on a iQ5 instrument (Bio‐Rad, Hercules CA, USA) in accordance with the manufacturer's instructions. Two micro liter of synthesized cDNA was used as template. Gene specific primers were designed using primer3plus, and maize actin 1 was used as a reference gene. All primers were presented in Supplemental Table S1.

## 3 RESULTS

### 3.1 Summary statistics and protein identification

**FIGURE 1:**
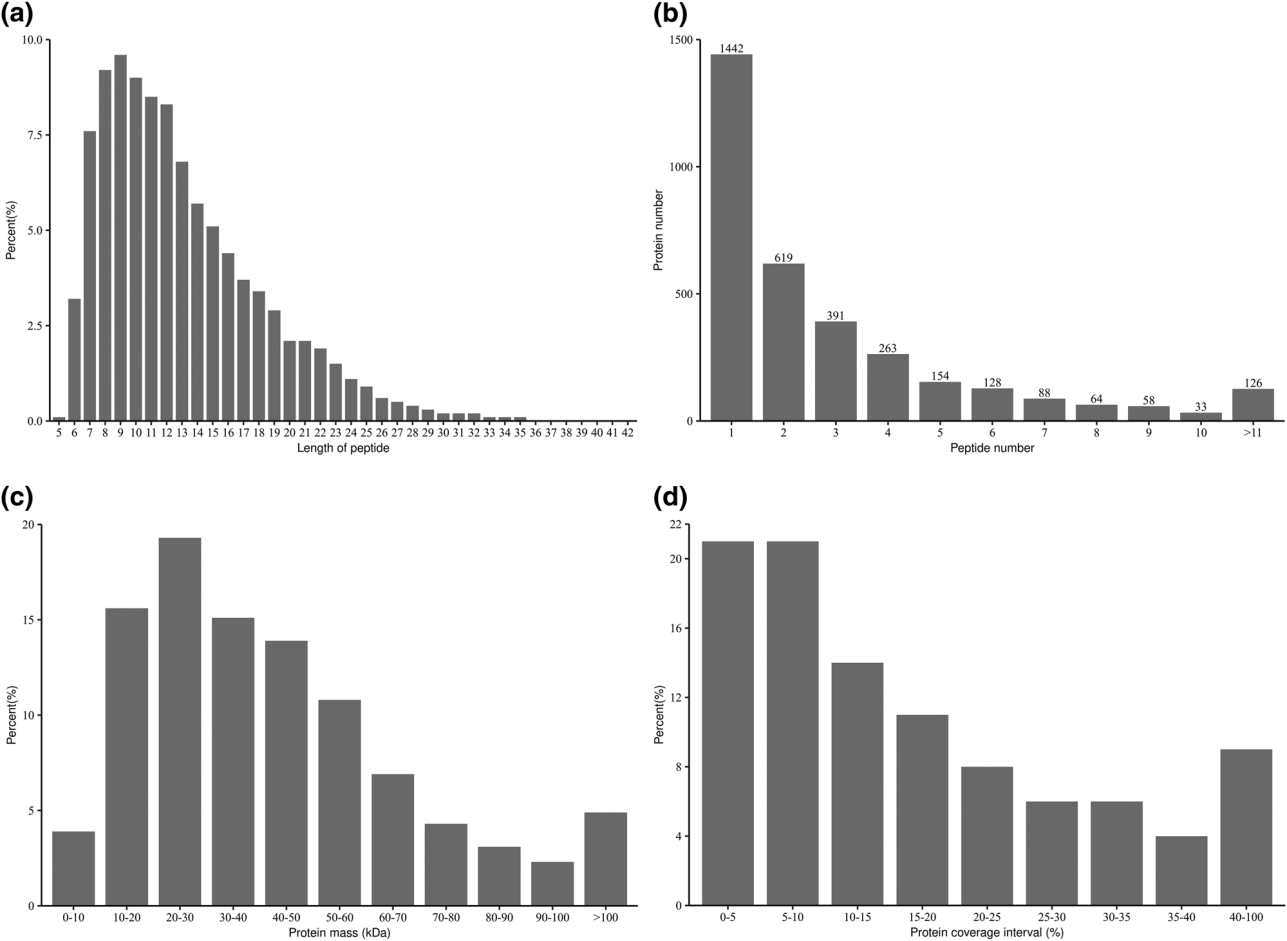
Distribution of length and number of peptides (a and b), mass of proteins and protein coverage interval (c and d) identified by iTRAQ proteomics

Using Mascot, we identified 14,582 unique peptides and 3,369 proteins from two biological replicates (Oberg & Vitek, 2009) in anthers of sterile, maintainer and restorer plants (Supplemental Tables S2 and S3). Peptide number and length distributions are shown in Figure 1a and 1b and protein masses and protein coverage interval are provided in Figure 1c and 1d. Among the identified proteins, 57.1% (1924) were inferred from more than two unique peptides (Figure 1a). Average predicted pIs and molecular weights of the identified proteins were 7.31 and 43.9 kDa, respectively. Protein coverage interval of 40–100%, 30–40%, 20–30%, 10–20%, and < 10% variation accounted for 9%, 10%, 14%, 25%, and 42% of identified proteins, respectively. Using iTRAQ strategy, 126 low‐molecular‐weight proteins (MW < 10 kDa) were identified, whereas no high‐molecular‐weight proteins (MW > 560 kDa) were detected.

To compare the relative abundance of the proteins in each sample type, we selected all identified proteins detected in two replicates and having at least two peptides. A total of 1,840 proteins meeting these criteria were used in the following differential accumulation analysis.

### 3.2 Identification of Differentially Accumulated Protein (DAP) species

**FIGURE 2:**
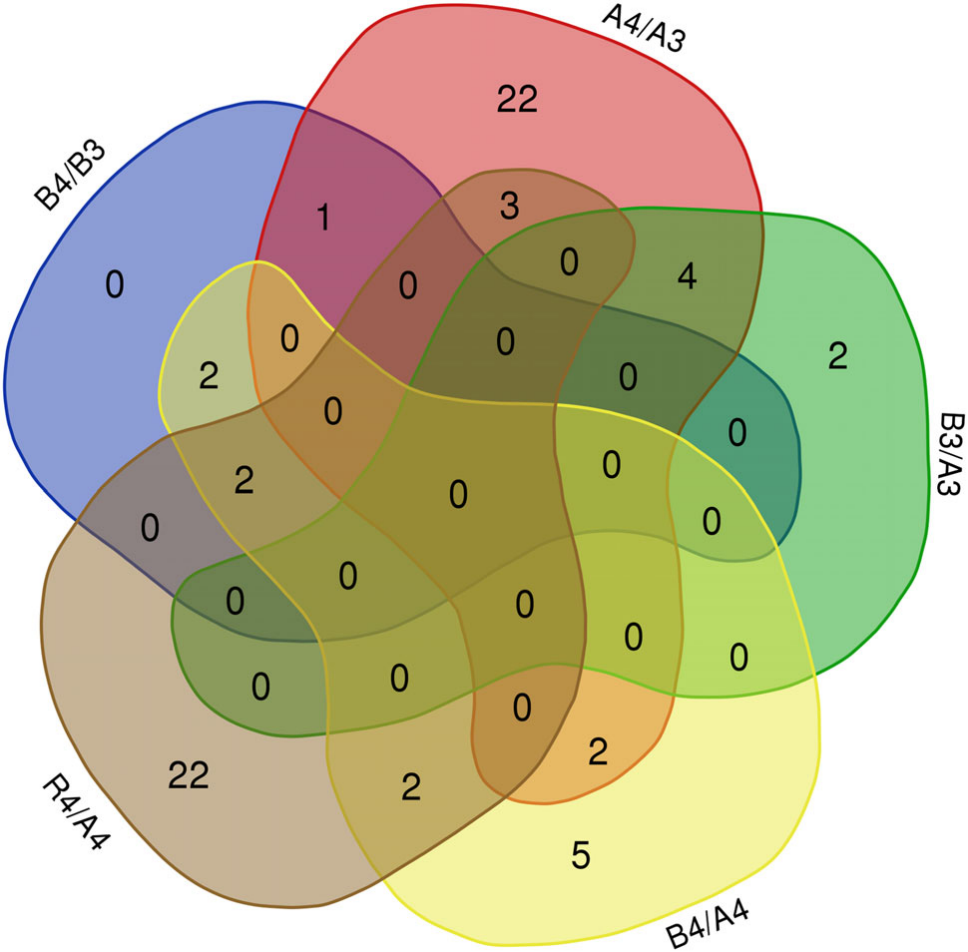
Venn diagram showing the comparative analysis of mitochondrial proteome of sterile, maintainer and restorer anthers A3, dyad stage of C^es^87‐1*^rfrf^*; A4, tetrad stage of C^es^87‐1*^rfrf^*; B3, dyad stage of N87‐1*^rfrf^*; B4, tetrad stage of N87‐1*^rfrf^*; R4, tetrad stage of C^es^87‐1*^Rf4Rf4^*

To identify and compare the sterility gene‐ and Rf gene‐responsive proteins and anther development proteins, we used the following criteria to designate significant differentially expressed proteins: expression fold‐change > 1.5‐fold and P‐value < .05 (Ting et al., 2009; Wang et al., 2016). In the five comparison groups, 70 proteins were classified as having a significant change in expression. Two chlorophyll related proteins were removed from subsequent analysis because of the possibility of a slight contamination possibly during the mitochondrial extraction process, and details of 68 genes was provided in Supplemental Table S4. Only one up‐accumulated protein was found from dyad to tetrad stages in anthers with normal cytoplasm, whereas the number of DAPs, especially up‐accumulated ones, was much higher in anthers with C cytoplasm (Figure 2). At dyad stage, six DAPs, five up‐regulated and one down‐regulated compared with A3, were detected. The number of DAPs between the maintainer line and the sterile line were increased rapidly at the tetrad stage compared with the dyad stage, which indicates that the sterility gene had finally exerted its detrimental effect on anther development. Under the background of the restorer gene, more DAPs were accumulated. Overall, low fold changes were observed among different comparisons. The highest and lowest fold change is 2.8 in R4 vs A4 and 0.4 in A4 vs A3, respectively.

### 3.3 Bioinformatic analysis of DAP species identified by iTRAQ

The identified proteins were first annotated automatically by automation pipeline tool against UniProt database, and the 68 DAPs were then carefully examined manually at Gramene maize annotation program (http://ensembl.gramene.org/Zea_mays/Info/Index). The results were summarized in Table 1 and Supplemental Table S4. In the comparison of B4 vs. A4, three DAPs were associated with the mitochondrial respiratory chain (Succinate dehydrogenase subunit 4, Cytochrome b‐c1 complex subunit 6 and ATP synthase subunit a, atp6); three were related to relief stresses caused by the sterile gene (rf2, ABC‐2 type transporter and Ubiquinol oxidase 1); four associated with energy metabolism (Fatty acyl‐CoA reductase, glucose translocator1, Lipolytic acyl hydrolase and Alanine aminotransferase 2) (Table 1). Whereas the R4 vs. A4 comparison showed that the DAPs only shared a small part. The restorer gene repressed expressions of DAPs in stress‐alleviation and in respiratory chain (although not significant) and increased protein accumulation in energy metabolism and protein processing (Table 1). DAPs in other comparisons generally fall into main functions mentioned above (Supplemental Table S4).

**TABLE 1:** Selected proteins showing differential accumulation between B4 and A4, and between R4 and A4

zm_gene_id	Cov(%)	Unique Peptides	B4/A4 ratio#	R4/A4 ratio	Description
Zm00001d048337	12.480	4	2.740	2.845	Fatty acyl‐CoA reductase; Male sterility, NAD‐binding; chloroplast
Zm00001d027837	31.310	14	1.477	2.076	Cytochrome P450‐like protein,ms26
Zm00001d028410	22.400	6	1.553	1.902	Glucan endo‐1,3‐beta‐glucosidase 7
Zm00001d024712	12.450	6	1.261	1.802	Flavonoid 3‐monooxygenase
Zm00001d047638	9.790	3	1.030	1.788	Calcium‐dependent phosphotriesterase superfamily protein
Zm00001d011932	9.370	3	1.285	1.778	Putative cytochrome P450 superfamily protein
Zm00001d034032	44.720	16	1.259	1.711	Strictosidine synthase involved in indole alkaloid biosynthesis
Zm00001d046537	8.580	2	1.639	1.663	ABC‐2 type transporter;SEC14‐like protein 1 isoform 2
Zm00001d021021	28.170	3	1.397	1.640	Peptidyl‐prolyl cis‐trans isomerase
Zm00001d013340	6.410	3	1.385	1.585	Nucleotide‐diphospho‐sugar transferase family protein
Zm00001d045042	20.070	12	0.982	1.584	Sucrose synthase 1
Zm00001d048079	4.580	2	1.410	1.573	Polygalacturonase
Zm00001d009858	32.530	13	1.290	1.555	Trans‐cinnamate 4‐monooxygenase
Zm00001d037590	48.630	14	1.272	1.539	Protein disulfide isomerase
Zm00001d040766	47.410	13	1.323	1.527	PDIL2‐2‐Zea mays protein disulfide isomerase
Zm00001d013282	34.880	3	1.508	1.524	40S ribosomal protein S27
Zm00001d049099	54.670	18	1.200	1.520	Protein disulfide‐isomerase
Zm00001d009908	19.260	2	1.309	1.514	UDP‐glucuronic acid decarboxylase 4
Zm00001d008567	11.530	2	1.587	1.465	glct1;glucose translocator1:[source:characterized gene
Zm00001d038481	5.860	2	1.647	1.434	Serine‐threonine/tyrosine‐protein kinase,catalytic domain
Zm00001d048841	37.800	2	1.553	1.419	Patatin T5,Lipolytic acyl hydrolase(LAH),Lipid degradation
Zm00001d009211	31.090	3	1.684	1.275	GTP‐binding protein SAR1A
Zm00001d045221	8.430	3	0.583	1.159	Thiol protease SEN102
Zm00001d034721	4.040	2	1.567	1.096	DEAD‐box ATP‐dependent RNA helicase
ZeamMp182	8.940	3	0.539	0.934	ATP synthase subunit a,atp6, mito
Zm00001d010491	11.420	2	0.582	0.843	Succinate dehydrogenase subunit 4 mito
Zm00001d016722	44.930	2	0.664	0.812	Cytochrome b‐c1 complex subunit 6
Zm00001d013785	21.510	3	0.611	0.803	Putative uncharacterized protein
Zm00001d002436	22.490	7	0.605	0.786	Ubiquinol oxidase 1;alternative oxidase 1
Zm00001d011135	15.290	3	0.593	0.745	Outer mitochondrial membrane protein porin
Zm00001d018639	31.170	3	0.651	0.690	Alanine aminotransferase 2
Zm00001d048324	25.610	4	0.828	0.667	Ribulose‐phosphate 3‐epimerase
Zm00001d011610	17.860	2	0.935	0.663	Glutamate synthase 1[NADH]chloroplastic
Zm00001d019243	26.840	5	0.769	0.647	Uncharacterized protein
Zm00001d045451	30.670	16	0.772	0.635	Transketolase 1,chloroplastic
Zm00001d012674	44.870	7	0.735	0.632	Chorismate mutase 3
Zm00001d011526	14.840	3	0.786	0.629	Ribokinase
Zm00001d045706	60.840	23	0.498	0.613	rf2,T‐cytoplasm male sterility restorer factor 2
Zm00001d042282	41.630	3	0.683	0.604	Transaldolase 2
Zm00001d012674	42.310	6	0.711	0.594	Chorismate mutase
Zm00001d041569	26.840	2	1.143	0.582	Translocon‐associated protein subunit beta
Zm00001d006182	6.000	2	0.755	0.575	aldolase like
Zm00001d006900	10.230	2	0.605	0.546	Phospho‐2‐dehydro‐3‐deoxyheptonate aldolase 2; DAHP synthetase

A3, dyad stage of C^es^87‐1*^rfrf^*; A4, tetrad stage of C^es^87‐1*^rfrf^*; B3, dyad stage of N87‐1*^rfrf^*; B4, tetrad stage of N87‐1*^rfrf^*; R4, tetrad stage of C^es^87‐1*^Rf4Rf4^*; #, fold change > 1.5 and *P*‐value < .05; Orange: up‐regulation, Light‐blue: down‐regulation; Cov: Coverage, indicates average percentage of assigned peptides to predicted protein.

Gene Ontology (GO) annotation was applied to further characterize significantly enriched functional groups of DAPs at different time points and in different samples. The results of GO term enrichment are shown in Supplemental Table S5. Sixteen DAPs (50%) between A4 and A3 were classified into 14 functional groups, all related to cellular components, except for two proteins, these annotated protein species were strongly enriched in mitochondria‐related terms, such as “mitochondrial membrane” (GO:0031966, p_adj_ = 0.01) and “mitochondrial protein complex” (GO:0098798, p_adj_ = 0.01) (Supplemental Table S5). No GO terms were enriched between B4 and B3, as only one protein was significantly differentially accumulated between these two timepoints.

Fourteen DAPs (66.7%) between B4 and A4 were classified into 18 GO subcategories. Under the biological process category, the most abundant DAPs were related to group II intron splicing and chorismate metabolic process. In regards to cellular components, DAPs were mostly classified into mitochondrial outer membrane and respiratory chain in cellular component subcategories. DAPs associated with molecular function were related to oxidoreductase and transaminase activities. DAPs between R4 and A4 were related to a wide range of functions, being grouped into 22 biological process, 2 cellular component and 8 molecular function subcategories (Supplemental Table S5). DAPs in this comparison group were mainly associated with isomerase, transferase and oxidoreductase activities and were mostly located in the endomembrane system. No enriched proteins were identified in the comparison between B3 and A3.

The main metabolic, synthetic and signal transduction pathways of the participating DAPs between A4 and A3, B4 and A4, and R4 and A4, respectively, were determined by the KEGG pathway enrichment analysis. The results of this analysis are presented in Supplemental Table S6. The most represented pathway during dyad to tetrad stages of CMS‐C anther development (A4 vs. A3) was fatty acid degradation, which was followed by AGE‐RAGE signaling and histidine metabolism; in contrast, no KEGG pathways were enriched in anthers with normal cytoplasm. At the tetrad stage, pathways enriched between N cytoplasm and C cytoplasm were mainly related to amino acid metabolism, especially amino acid biosynthesis, pentose phosphates and cutin, suberin and wax biosynthesis.

### 3.4 Localization validation of DAPs through bioinformatic prediction

To verify if the 68 DAPs were actually targeted to mitochondrion, the full protein sequences were extracted and subjected to signal peptide or subcellular localization prediction by SignalP (www.cbs.dtu.dk/services/SignalP/), TargetP (www.cbs.dtu.dk/services/TargetP/), WoLFPSORT (www.genscript.com/wolfpsort.html) and UniProt (www.uniprot.org/). The results showed that 21 (31.9%) DAPs were located to the mitochondrion, 29 (42.6%) to the chloroplast and 9 (25.5%) to other subcellular compartments (Table 2). Maize anthers grow nearly in darkness and only endothelial cells show a low density of chloroplasts (Murphy, Egger, & Walbot, 2015). Thus, we suspected that these over half non‐mitochondrial DAPs were still transported to mitochondrion. To test this, we compared 29 predicted chloroplast‐targeted DAPs with 239 maize proteins expressed in the maize leaf and related to photosynthesis (Murphy et al., 2015) and found that only two DAPs, Zm00001d045451 and Zm00001d048324, were included in this maize leaf protein set. The other DAPs, including 21 to mitochondrion, were all excluded from the leaf protein set. Although these two DAPs are expressed in maize leaf and 9 DAPs were predicted to be sorted to other sub‐cell locations, they are probably also transported to mitochondrion, because many proteins are proposed to be dual‐targeted (Carrie & Small, 2013). Together, these results demonstrate, to some extent, that the identified proteins are truly mitochondrion‐targeted.

**TABLE 2:** Summary of localization verification through prediction tools

Gene ID	TargetP/SignalP^a^	Uniprot	WoLFPSORT	Summary^b^
ZeamMp182	Other	Mitochondrion	Mitochondrion	M
Zm00001d002246	Other	Peroxisome	Endoplasmic reticulum	O
Zm00001d002436	Mitochondrion	Other	Chloroplast	M
Zm00001d003086	Chloroplast	No Results	Chloroplast	C
Zm00001d003702	Other	No Results	Mitochondrion	M
Zm00001d006182	Chloroplast	Other	Chloroplast	C
Zm00001d006900	Mitochondrion	Chloroplast	Chloroplast	M
Zm00001d006914	Other	Nucleus/Other	Cytoplasmic	C
Zm00001d007153	Other	Mitochondrion	Chloroplast	M
Zm00001d008567	Chloroplast	Other	Plasma membrane	C
Zm00001d009211	Other	Endoplasmic reticulum/Other	Chloroplast	C
Zm00001d009858	Signal peptide	Other	Cytoplasmic	O
Zm00001d009908	Other	Other	vacuolar	O
Zm00001d010491	Mitochondrion	No Results	Mitochondrion	M
Zm00001d010731	Mitochondrion	Mitochondrion	Chloroplast	M
Zm00001d011135	Other	Mitochondrion	Cytoplasmic	M
Zm00001d011526	Chloroplast	Nucleus/Other	Mitochondrion	M
Zm00001d011610	Other	No Results	Cytoplasmic	O
Zm00001d011932	Signal peptide	Other	Chloroplast	C
Zm00001d012599	Mitochondrion	Mitochondrion	Chloroplast	M
Zm00001d012674	Chloroplast	Other	Chloroplast	C
Zm00001d013282	Other	Other	Chloroplast	C
Zm00001d013340	Signal peptide	No Results	Chloroplast	C
Zm00001d013785	Mitochondrion	No Results	Chloroplast	M
Zm00001d015127	Mitochondrion	No Results	Chloroplast	M
Zm00001d016274	Other	Other	Plasma membrane	O
Zm00001d016722	Other	Mitochondrion	Chloroplast	M
Zm00001d017768	Other	Other	Nucleus	O
Zm00001d018639	Other	No Results	Chloroplast	C
Zm00001d019045	Other	Nucleus/Other	Nucleus	O
Zm00001d019243	Chloroplast	No Results	Chloroplast	C
Zm00001d019600	Other	Other	Chloroplast	C
Zm00001d020727	Other	No Results	peroxisome	O
Zm00001d020794	Signal peptide	Plasma Membrane	Extracellular	O
Zm00001d021021	Signal peptide	No Results	Extracellular	O
Zm00001d021804	Mitochondrion	No Results	Chloroplast	M
Zm00001d023220	Other	Mitochondrion	Cytoplasmic	M
Zm00001d024712	Other	Other	Chloroplast	O
Zm00001d027837	Other	cellular component	Chloroplast	C
Zm00001d028410	Signal peptide	Plasma Membrane	Chloroplast	C
Zm00001d031942	Other	Endosome/Other	Chloroplast	C
Zm00001d031956	Other	Other	Cytoplasmic	C
Zm00001d033062	Other	No Results	Mitochondrion	M
Zm00001d034032	Signal peptide	Endoplasmic reticulum	Chloroplast	C
Zm00001d034721	Chloroplast	Chloroplast	Chloroplast	C
Zm00001d034793	Other	Mitochondrion	Mitochondrion	M
Zm00001d036417	Mitochondrion	Mitochondrion	Chloroplast	M
Zm00001d037590	Signal peptide	Endoplasmic reticulum	Chloroplast	C
Zm00001d038481	Signal peptide	Endoplasmic reticulum/Other	Plasma membrane	O
Zm00001d039656	Mitochondrion	Mitochondrion/Other	Mitochondrion	M
Zm00001d040766	Signal peptide	Endoplasmic reticulum/Other	Extracellular	O
Zm00001d041569	Signal peptide	Endoplasmic reticulum	Chloroplast	C
Zm00001d042282	Chloroplast	Other	Chloroplast	C
Zm00001d045042	Mitochondrion	No Results	Cytoplasmic	M
Zm00001d045221	Signal peptide	Extracellular or secreted	Chloroplast	C
Zm00001d045251	Other	Peroxisome	Chloroplast	C
Zm00001d045451^c^	Chloroplast	Cytosol	Chloroplast	C
Zm00001d045706	Mitochondrion	No Results	Mitochondrion	M
Zm00001d046120	Other	No Results	Chloroplast	C
Zm00001d046537	Other	Plasma Membrane	Plasma membrane	O
Zm00001d046591	Other	Other	Plasma membrane	O
Zm00001d047638	Signal peptide	Endoplasmic reticulum/Other	Chloroplast	C
Zm00001d048079	Signal peptide	Extracellular or secreted	Extracellular	O
Zm00001d048324^c^	Chloroplast	Cytosol	Chloroplast	C
Zm00001d048337	Chloroplast	Chloroplast	Chloroplast	C
Zm00001d048841	Other	No Results	Chloroplast	C
Zm00001d049099	Signal peptide	Other	vacuolar	O
Zm00001d053899	Other	Cytoskeleton	Plasma membrane	O

^a^Results from two tools, TargetP and SignalP.

^b^Final localization of gene; M: mitochondrion, C: chloroplast, O: other location.

^c^Expressed chloroplast genes detected by microarray (Ma, Skibbe, Fernandes, & Walbot, 2008).

### 3.5 Expression comparison between RNA and protein

**FIGURE 3:**
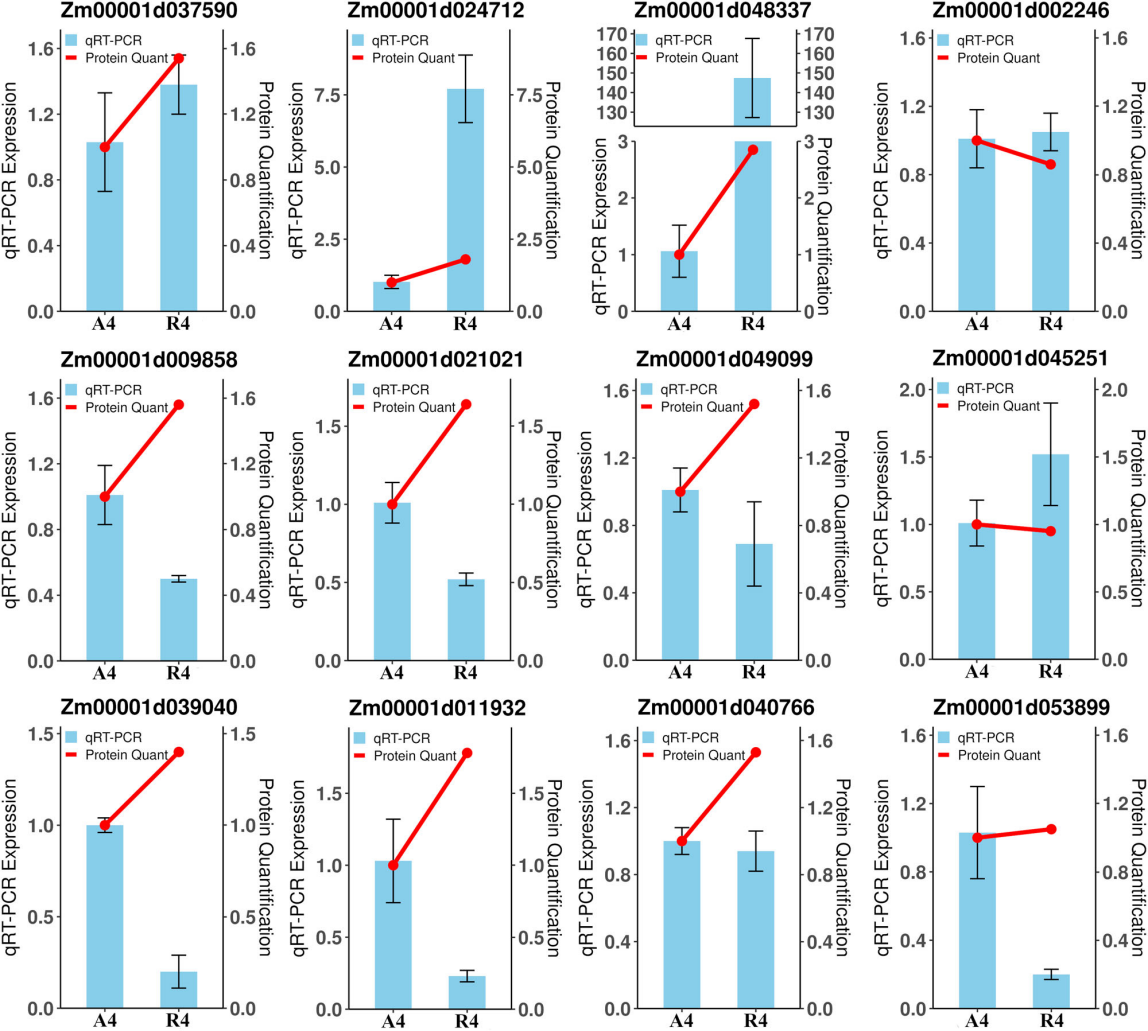
Experimental validation of 12 differentially accumulated proteins in the RNA level using qRT‐PCR. A4, tetrad stage of C^es^87‐1*^rfrf^*; R4, tetrad stage of C^es^87‐1*^Rf4Rf4^*; The red lines with dots on two sides represent the protein expression trend between two materials

To check RNA expression levels by qRT‐PCR, we selected 12 DAPs involved in fatty acid degradation, amino acid metabolism and protein maturation pathways. Except for Zm00001d002246 and Zm00001d040766 (Figure 3), each gene was differentially expressed at both RNA and protein levels, but their RNA and protein expression tendencies differed greatly. Three genes were up‐regulated at both RNA and protein level; two of these (especially Zm00001d048337) had a much higher expressions at the RNA level and were predicted to be involved in the fatty acid degradation pathway. Five genes (Zm00001d009858, Zm00001d021021, Zm00001d049099, Zm00001d039040 and Zm00001d011932) were significantly accumulated at the protein level (Figure 3; Supplemental Table S4), whereas the corresponding RNAs were significantly decreased at the same developmental stage. Zm00001d040766 was only significantly expressed at the protein level. Genes related to protein maturation, namely, Zm00001d021021 encoding a peptidyl‐prolyl cis‐trans isomerase and Zm00001d037590, Zm00001d040766 and Zm00001d049099 encoding protein disulfide isomerase, respectively, were all up‐accumulated at the protein level.

## 4 DISCUSSION

The CMS system is an efficient, economical tool for exploitation of heterosis by maize breeders. Although maize CMS‐C has been widely used in hybrid seed production, and extensive research involving segregating population (Kohls et al., 2011; Tang et al., 2001), genomics (Allen et al., 2007), epigenetics (Chen et al., 2016; Li et al., 2017a), transcriptomics (Li et al., 2017b; Liu et al., 2018; Xue et al., 2019), has been conducted, the molecular mechanism of sterility and restoration in CMS‐C maize remains to be further explored.

Proteins are the main actors in biological activities. Expression levels of proteins are better predictors for revealing the mechanism of CMS than those of mRNAs because protein levels are not completely correlated with transcript abundance (Vogel & Marcotte, 2012). The results of our comparison of relative expression levels of several genes inferred from RNAs vs. their proteins are consistent with the above conclusion (Figure 3). The causal gene of CMS is well known to be present in DNA from organelles such as mitochondria, while the corresponding restorer, although nuclear in origin, must function in organelles as well. Mitoproteomics is therefore a useful approach for uncovering complex changes in maize anthers in isonuclear‐alloplasmic and isoplasmic‐near‐isogenic lines and can provide novel information concerning sterility and restoration in CMS‐C maize.

In this study, iTRAQ quantitative proteomic technology was applied to analyze mitochondrial‐protein expression profiles of maize anthers at dyad and tetrad stages during meiosis. A total of 68 DAPs were identified by comparative proteomic analysis (Table 1; Supplemental Table S4). The distribution of these DAPs at the different developmental stages indicates that the detrimental effect of the sterility gene is manifested after the dyad stage (Figure 2). Bioinformatics analysis uncovered a complex metabolic network that includes fatty acid degradation, amino acid biosynthesis, the pentose phosphate pathway, protein processing in the endoplasmic reticulum and AGE‐RAGE signaling. These metabolic pathways might be associated with mechanisms underlying the abortion of pollen development by the potential sterility gene and the compensation or reversal of these processes by the nuclear restorer.

### 4.1 DAPs related to fatty acid degradation

Fatty acids, which are degraded and oxidized only in the mitochondria and peroxisomes, are important energy sources for cell activities. NADH and FADH2 generated by both fatty acid β‐oxidation and the TCA cycle are used by the electron transport chain to produce ATP. Oxidation of fatty acids can produce 33% more amounts of energy (ATP) than that obtained from glucoses (Goetzman, 2011). Variation in ATP content can affect plant growth and development (Tiwari, Belenghi, & Levine, 2002). Although energy requirements increase dramatically during the reproductive stage, especially during anther development (Li, Zhang, Xia, Deng, & Ye, 2010), the sterility factor perturbs the normal function of the respiratory electron transfer chain and hinders the sufficient production of ATPs. The structure and function of F_1_F_0_‐ATPase is affected by the CMS‐related gene *atp6‐orfH79* in HL‐CMS rice (Wang et al., 2013; Zhang et al., 2007). Changes in *atpA* expression in the sesame CMS line reduced ATP biosynthesis in the mitochondria (Yang et al., 2008). In the SaNa‐1A CMS line of *Brassica napus*, ATP synthesis is inhibited by the abnormal structure and function of terminal oxidase caused by the sterility factor (Du et al., 2019). The fatty acid oxidation pathway is therefore initiated to satisfy the energy demand. In our study, two proteins involved in fatty acid oxidation (Zm00001d045251 and Zm00001d045706) were identified, but no expression difference was detected at the dyad stage between normal and the C cytoplasm (Figure 2; Supplemental Table S4). Their expression levels significantly increased from dyad to tetrad stages in C cytoplasm compared with normal cytoplasm, which suggests the occurrence of an ATP deficiency in C cytoplasm at the second phase of meiosis during anther development. Measurement of ATPase activities has confirmed such a deficiency, and with introduction of the restorer gene, it is able to restore the ATP content to a level sufficient to meet the energy requirements of anther development (Xue et al., 2019). Consistent with this report, one of the proteins (Zm00001d045706) was significantly decreased at the tetrad stage in the restorer lines (Figure 2). Significant expression differences of fatty acid metabolism‐related genes were also detected in a transcriptome analysis of CMS lines of maize and other species (Li et al., 2017b; Lin et al., 2019; Xue et al., 2019). In contrast to genes detected by transcriptome analysis, which are mainly present in cytosol, genes detected in the present study function in mitochondria, where they are involved in the TCA cycle and are coupled to the electron transport chain.

### 4.2 DAPs related to amino acid metabolism

Amino acids are not only fundamental components in protein synthesis; they also play essential roles in signaling processes and stress response. In humans, errors in amino acid metabolism can lead to a series of disorders, such as phenylketonuria, tyrosinemias and hyperprolinemia (DeArmond, Dietzen, & Pyle‐Eilola, 2017; Manoli & Venditti, 2016). In plants, amino acids can directly or indirectly influence a plethora of physiological activities, such as biotic and abiotic stress resistance (Rai, 2002), growth and development (Watanabe et al., 2013), and signaling (Häusler, Ludewig, & Krueger, 2014; Hildebrandt, Nunes Nesi, Araújo, & Braun, 2015; Pratelli & Pilot, 2014). Under unfavorable cell condition, amino acids, which are used as an alternative respiratory substrates, can be metabolized via the TCA cycle to meet the energy shortages (Araújo, Tohge, Ishizaki, Leaver, & Fernie, 2011; Galili, Avin‐Wittenberg, Angelovici, & Fernie, 2014; Watanabe et al., 2013). In our study, Zm00001d045706, a protein related to the degradation of three branched‐chain amino acids (BCAAs), Val, Leu and Ile, accumulated significantly (2.5 times) from the dyad to tetrad stage in the sterile lines—an indirect manifestation of energy deficiency. In the maintainer line and the restorer line, the accumulation of this protein was only slightly elevated and this change was not significant. The BCAA degradation pathway was enriched between A4 and A3 and between B4 and A4, but not between R4 and A4; this result indicates that the energy shortage was compensated under the background of the restorer gene. Enzymes (proteins) involved in the aromatic amino acids synthetic pathways also accumulated, especially seven proteins identified in the comparison between R4 and A4 (Table 1). Aromatic amino acids are precursors of several plant hormones, such as auxin, salicylate acid and anthocyanin (Tzin & Galili, 2010; Tzin, Galili, & Aharoni, 2012), that may promote microspore maturation. The decrease of these seven proteins at the tetrad stage of the restorer line (R4) indicates that the anther development in the sterile lines was disrupted or delayed.

### 4.3 DAPs involved in protein maturation

Proper processing of proteins after translation is essential for the correct functioning of living cells, and misfolding of a protein can cause severe disease. The most prominent example of misfolded proteins are prions, which are responsible for the notorious mad cow disease (Greenlee & Greenlee, 2015; Lee, Kim, Hwang, Ju, & Woo, 2013). Several chaperones act together for either refolding or degradation of a target protein (Pulido et al., 2016). In the comparison of R4 and A4 in our study, four genes were found to be significantly accumulated; a peptidyl‐prolyl cis‐trans isomerase (PPIase) and three protein disulfide isomerases (PDI) (Table 1; Supplemental Table S4). PPIases catalyze the cis/trans conformational transition of the peptidyl‒prolyl peptide bond in proteins, that facilitates correct protein folding (Ahmad et al., 2019; Bissoli et al., 2012; Gavini, Tungtur, & Pulakat, 2006), while PDIs repaires uncorrected disulfide arrangements (Ali Khan & Mutus, 2014; Okumura et al., 2019; Wilkinson, 2004). Noteworthy, PPIases can improve the efficiency of PDIs in protein folding (Schonbrunner & Schmid, 1992). Peptides specific to the putative sterility gene compared with the normal *atp6* gene (Allen et al., 2007) may affect the efficiency of its folding or the correct conformation, which reduces the final amount of the ATPase complex, thereby leading to energy reduction and sterility. In the restorer line, the restorer gene up‐regulates the gene related to protein maturation and restores fertility.

## 5 CONCLUSIONS

Because information about the sterility gene and corresponding restorer gene(s) in CMS‐C maize is lacking, the mechanism of sterility and fertility restoration in this species is still unclear. Taking into account that factors responsible for CMS are located in mitochondrial DNA, we thus focused on changes within mitochondria in the CMS‐C maize system during anther development using high‐throughput proteomics technology. Our comparison of proteins differentially expressed between CMS and maintainer lines supported the energy deficiency hypothesis for CMS‐C maize. Further comparison between CMS and restorer lines suggested that the restorer gene not only initiates alternative metabolic pathways to meet energy requirements, but also improves the efficiency of post‐translation processing of the unidentified sterility factor to promote its maturation and assembly in an electronic transport complex. These results, which provide more details on restoration and sterility mechanism, should advance the cloning of restorer genes and sterility factors.

## CONFLICT OF INTEREST STATEMENT

The authors declare that there is no conflict of interest.

## AUTHOR CONTRIBUTIONS STATEMENT

XY and TJ designed the research; ZH, WB, LY and LB performed the experiments; ZH, YH and LB analyzed data; XY, DD and TJ wrote the manuscript.

## Supporting information

Supplemental Table S1: Primers used for QPCR detection.

Supplemental Table S2: peptide identification and quantification

Supplemental Table S3: protein identification and quantification

Supplemental Table S4: Significantly differentially‐accumulated proteins in five comparisons

Supplemental Table S5: GO enrichment of different comparisons

Supplemental Table S6: KEGG enrichment of different comparisons
